# Creating a Successful Virtual Reality–Based Medical Simulation Environment: Tutorial

**DOI:** 10.2196/41090

**Published:** 2023-02-14

**Authors:** Sanchit Gupta, Kyle Wilcocks, Clyde Matava, Julian Wiegelmann, Lilia Kaustov, Fahad Alam

**Affiliations:** 1 Department of Anesthesia Sunnybrook Health Sciences Centre Toronto, ON Canada; 2 Temerty Faculty of Medicine University of Toronto Toronto, ON Canada; 3 Department of Anesthesia and Pain Medicine Hospital for Sick Children Toronto, ON Canada; 4 Department of Anesthesia and Pain Medicine University of Toronto Toronto, ON Canada

**Keywords:** virtual reality, innovation, digital health, simulation, medical education, medical training, tutorial, how-to, curriculum

## Abstract

Innovation in medical education is not only inevitable but a requirement. Manikin-based simulation is currently the gold standard for supplemental clinical training; however, this modality requires significant equipment and personnel to operate. Virtual reality (VR) is emerging as a new method of delivering medical simulation sessions that requires less infrastructure but also allows for greater accessibility and flexibility. VR has slowly been integrated into the medical curriculum in some hospitals; however, more widespread adoption would transform the delivery of medical education for future clinicians. This tutorial introduces educators to the BUILD REALITY (begin, use, identify, leverage, define, recreate, educate, adapt, look, identify, test, amplify) framework, a series of practical tips for designing and implementing a VR-based medical simulation environment in their curriculum. The suggestions are based on the relevant literature and the authors’ personal experience in creating and implementing VR environments for medical trainees. Altogether, this paper provides guidance on conducting a needs assessment, setting objectives, designing a VR environment, and incorporating the session into the broader medical curriculum.

## Introduction

Medical education is transforming. Currently, manikin-based simulation is the gold standard used for clinical training, yet, despite being effective, it is quite resource-intensive. Manikin-based simulation requires dedicated space, equipment, and personnel to run simulation sessions for medical trainees [1,2]. Often, the educational facility will need simulation specialists to oversee the simulation and medical facilitators to debrief participants to support learning.

Virtual reality (VR) is emerging as a new, flexible method of delivering simulation sessions that allows for educational standardization. Central to VR is the concept of immersion, which is defined as the perception and belief of being present in a simulated world [3]. VR is a computer-generated world that involves immersion and sensory feedback. VR-based medical simulation offers benefits for both medical learners and educators by providing various means of delivering learning content [3-5]. VR is standardized, accessible, and can have assessment metrics and feedback built into the VR environment. Moreover, the medical trainee can go through the VR environment remotely, at any location or time of day. VR allows learners to make mistakes safely and then learn through deliberate practice to improve their performance without harming any patients [6].

The successful application of VR in medical education requires careful planning and implementation. Through our experience launching VR-based clinical simulation sessions in hospitals such as the Sunnybrook Health Sciences Centre, the Hospital for Sick Children, and the Sunnybrook Canadian Simulation Centre, this tutorial aims to provide educators with a series of practical suggestions for designing and implementing VR-based medical education sessions (Textbox 1). Throughout this paper, we will outline the BUILD REALITY (begin, use, identify, leverage, define, recreate, educate, adapt, look, identify, test, amplify) framework and use our experience from the development and implementation of our VR environment as a case study to further reinforce our suggestions. The VR-based medical simulation environment we developed is (1) being used in the Sunnybrook Simulation Centre and (2) being tested in a clinical trial (Clinicaltrials.gov NCT04451590) to assess whether it can enhance the decision-making skills of medical trainees during an airway injury crisis scenario (Multimedia Appendix 1).

**The BUILD REALITY (begin, use, identify, leverage, define, recreate, educate, adapt, look, identify, test, amplify) framework for designing and implementing a virtual reality–based medical simulation environment.**

**Design**

- Begin with a needs assessment
- Use the needs assessment to set objectives
- Identify the best virtual reality (VR) modality
- Leverage and build content based on learning theory
- Define and support the cocreation of the VR environment
- Recreate diversity and accessibility within the VR environment

**Implementation**

- Educate users with a prebriefing
- Adapt and test the VR environment with learners and educators
- Look for VR simulation champions
- Identify barriers
- Test the impact of the VR tool
- Amplify VR in the 21st century: value within the broader curriculum

### Design

#### Begin With a Needs Assessment

Before creating a new VR clinical environment, it is important to involve all stakeholders and conduct a needs assessment. The stakeholders that should be involved include the end users, human factor specialists, content experts, and software design technical experts. The team should conduct interviews, use focus groups, and make real-life observations to identify an unmet problem in the medical education system.

**Figure 1:**
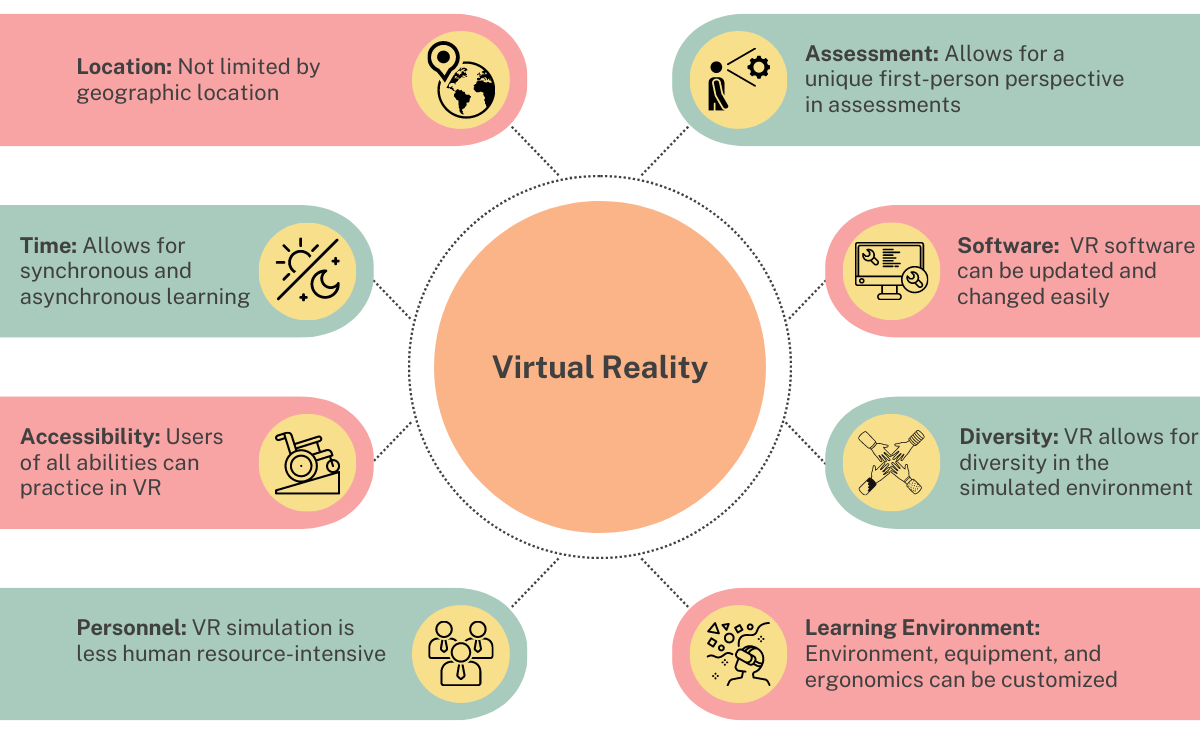
Factors to consider in a needs assessment that promote the use of virtual reality. VR: virtual reality.

As shown in Figure 1, there are certain factors to consider in a needs assessment that may promote creating a VR-based medical environment over another teaching modality. These factors include location, time, accessibility, assessment, personnel, software, diversity, and learning environment [4-6]. Compared to manikin simulation, VR simulation is not geographically constrained and allows for asynchronous learning. VR environments can be designed to be accessible to the user, especially for individuals with mobility constraints, and they require less intensive use of hospital and human resources than manikin simulation. Compared to other teaching modalities, the learning-by-doing nature and first-person perspective of VR allows for new forms of assessment and evaluation. The VR environment can easily be updated and changed as new medical guidelines are released and diversity can be built in through various avatars and virtual patients. Finally, the learning environment can be customized to replicate any environment (eg, an operating room or a trauma center), including simulated equipment and ergonomics.

If the needs assessment identifies a gap requiring a standardized, accessible, or self-regulated solution, then VR is an up-and-coming technological solution [6]. In the past, VR environments have been created for procedure education support, anatomy training, and clinical decision-making. VR can be used to educate patients, medical students, residents, other health care providers, and interprofessional teams [7-10]. Before creating a VR environment, one should perform thorough market research to see if another laboratory or commercial entity has already created a VR environment that satisfies the educational requirements. If this is the case, the VR assets or environment can be shared and downloaded onto the VR platform used. If it is decided that a VR environment should be developed, budget support should be considered for both the technical and nontechnical expenses of the project.

As part of the needs assessment for our VR airway scenario, we collected feedback through focus groups from various program directors, nurses, medical learners, trauma physicians, and anesthesiologists. Additionally, we conducted clinical observations of manikin-based simulation sessions and real-life airway trauma cases to identify gaps that could be addressed through VR.

#### Use the Needs Assessment to Set Objectives

The objectives should be aligned with an education evaluation model, such as the Kirkpatrick model [11]. The Kirkpatrick model is used to evaluate the effectiveness of a learning program and allows for objective setting early in the development pipeline. For instance, with a VR-based simulation environment, the Kirkpatrick model objectives related to the anticipated reaction, learning, behavior, and results [12] should be set out during the design stage of the VR environment. With these objectives in mind, the team can work to select certain parameters, such as the type of VR headset and environment. The objectives should be aligned with the latest medical textbooks and reviewed with stakeholders and end users. In helping to formulate the objectives, one should involve an interprofessional group of educators—this ensures all professional perspectives can be drawn upon [13]. Based on the use case of the VR environment (eg, memorization or decision-making), the group should set objectives based on knowledge acquisition, application, and core decision-making steps that need to be conveyed.

For instance, for our airway crisis management scenario, we created objectives related to the content, technical skills, and nontechnical skills that needed to be conveyed (Multimedia Appendix 2).

#### Identify the Best VR Modality

Once the needs assessment and the objectives are set, the interprofessional team should determine the level of immersion, interactivity (passive vs active), and the modality required for the environment. It should be noted that immersion can include sound, eye tracking, VR controllers, and haptic feedback, among other features. Interactivity in VR is often on a spectrum where passive VR is similar to watching an engaging movie and active VR is when one can manipulate an environment, similar to our airway environment (Multimedia Appendix 1). Once these parameters are decided upon, the hardware can easily be selected. The options include a screen-based or a stand-alone VR headset (Table 1) [14,15].

Based on our airway crisis scenario needs assessment and objectives, we wanted an immersive and active environment that simulated a trauma bay. Therefore, we used a stand-alone VR headset with sound, eye tracking, and controllers to allow learners to make decisions and physically practice their clinical decision-making.

**Table 1:** Comparison of screen-based and stand-alone virtual reality headsets.

	Screen-based virtual reality	Stand-alone virtual reality
Description	Interacting with a computer monitor, a smartphone, or a smartphone inside a lightweight, portable headset	Stand-alone headset with integrated processors and sensors
Price range	Low	Medium-high
Immersivity	Low-medium	Medium-high
Resolution	Low-medium	High
Motion tracking	Low	High
Equipment examples	Computer-based games, YouTube 360, Google Cardboard	Oculus Quest, Pico 4, HTC Vive Pro

#### Leverage and Build Content Based on Learning Theory

VR can simulate environments that enhance learning while also being interactive and immersive. To maximize the effectiveness of the VR environment, it should be built on sound learning theories, such as constructivism and self-regulated learning. For example, with constructivism, knowledge is constructed in a learning-by-doing fashion. Therefore, a VR-based simulation that allows the trainee to actively participate in the environment through navigation and manipulation is extremely beneficial [16].

An advantage of VR compared to manikin-based simulation is that it can be performed without access to a simulation center, which requires specialized personnel. VR is a modality that could provide a lower cost of learning where assessment and feedback processes can be preprogrammed into the VR environment and thus promote self-regulated learning [17]. This aspect ensures that the learner can go over key concepts at their own speed and practice as many times as needed [18]. The LOOP (learning theory, objectives, outcome, and output) framework is a design framework used for immersive VR environment development that is based on sound learning theory and objectives to create the VR output [19].

In our VR environment, the medical trainee goes through the core decision-making steps in an airway trauma to save a patient. While our scenario requires rapid decision-making, which presents a challenge for medical trainees, the trainee can go through the scenario as many times as needed. Each time, the algorithm provides feedback to promote self-regulated learning. Overall, we built the VR environment following self-regulated learning and constructivism learning theories.

#### Define and Support the Cocreation of the VR Environment

Cocreation occurs when learners and educators work collaboratively with one another to create educational resources [20]. An interprofessional team must be established to use the objectives to create a suitable VR environment. This will include individuals previously involved in the needs assessment and additional software developers, animators, human factor specialists, medical education researchers, and clinicians [21]. Together, they will provide the software background, curriculum content, and educational design input needed to effectively achieve the project outcomes. Any team must clearly define research questions, identify roles and responsibilities, set attainable goals, and communicate frequently.

The interdisciplinary team should follow three steps: (1) Create an outline, program goal, and detailed flowchart for the VR program. This skeleton should then link key educational goals and objectives with the visual elements in VR. (2) Use the outline as a building block for the developers and animators to create the first prototype of the VR environment. They will create these assets themselves or purchase assets. A game engine such as Unity or Unreal should be used when bringing together the assets, 3D models, 2D graphic designs, video elements, and voices [22]. (3) Test the initial iterations meticulously and evaluate both the VR environment and its use by learners; this is important in the design process.

We brought together an interprofessional team for our VR airway scenario, including software developers, learners (first to third years of medical education), program directors, medical educators, and health care professionals. A flowchart for the VR airway scenario is provided in Multimedia Appendix 3.

#### Recreate Diversity and Accessibility Within the VR Environment

Creating a new VR environment for the medical curriculum is a great opportunity to uphold the medical community’s commitment to inclusion, diversity, and equity. This can be done by creating patient and clinician avatars with diverse characteristics, such as age, height, weight, race, ethnicity, sex, gender, and health conditions. Since the VR environment can be repeated with ease, different patient or clinician avatars can be introduced in the medical trainees’ simulation curriculum. This opportunity for diversity is unique to VR when compared to traditional simulation-based medical education, where the purchased manikin is of the same sex and skin color for all medical trainees [23].

Additionally, VR allows the user to interact with the environment in multiple different ways. Users can teleport across the virtual room with a controller instead of walking, which is extremely beneficial for people who have physical disabilities. The room scale can be adjusted to eye height for individuals who need to be seated or are in a wheelchair [24]. These inherent accessibility elements should be introduced in the design of the VR environment to allow for increased utility.

In our case, the VR airway scenario included diverse avatars and various built-in features for accessibility needs. For instance, the medical trainee could use the controller to teleport across the trauma bay instead of walking, and they could move the virtual hospital bed up or down based on their height and reach (Multimedia Appendix 1).

### Implementation

#### Educate Users With a Prebriefing

Prebriefing is extremely important for both manikin and VR-based modalities. With manikin-based simulation, the facilitator summarizes the objectives of the environment, orients the participant to the environment, and provides a clear description of the participant’s role in the scenario [25]. Through VR, the prebriefing can be embedded within the VR environment as an acclimation room to avoid the need for specialized personnel and resources.

For many medical trainees and even educators, it could be their first time going through a VR environment. Therefore, the prebriefing session should include orientation for both technology and objectives. For example, once the headset is turned on, the orientation session should include how to navigate in the VR environment, how the hand tracking or controllers are used, and which objects can be manipulated.

For the VR airway environment, we prebriefed the objectives beforehand through email with the medical trainees. The technology prebriefing was delivered entirely through a VR acclimation room where the user was shown how to teleport in the virtual trauma bay and how to use the controllers to manipulate certain objects.

#### Adapt and Test the VR Environment With Learners and Educators

Once the prototype of the VR software is created, it should be piloted with the end user to receive feedback on content validity and VR usability. Effective usability testing does not have to be burdensome; typically, 5 to 6 sessions for any type of user is enough to reveal 95% of usability issues [26]. This process will help identify and resolve any errors. The entire setup should be tested at this stage, as follows: (1) Pre-VR: this stage includes selecting a designated VR area (eg, hospital, examination room, or home), setting up the VR equipment, introducing the technology to the users, and providing a prebriefing on how to navigate through the VR environment. (2) During VR: for immersive headsets, it is important to ensure that the user can teleport if they are in a large room or have enough space if they are walking around. The audio should be tested, and the environment should be clearly visible. It is also important for members outside the medical community, including developers and animators, to test the VR environment. All areas of the VR environment should be viewed and explored to uncover any problems. (3) Post-VR: a cleaning protocol should be determined for the VR headset and other equipment. Multiple options exist, including VR ultraviolet cleaning boxes and disinfectant wipes compatible with the brand of the VR headset. Logistics should be considered; for example, where the headset will be stored, how medical trainees can access the headset, if personnel are needed at the hospital, and if the trainees can take the equipment home.

Similar to manikin-based simulation, validity can be assessed through a pretest followed by a training session and a posttest. Furthermore, an independent rater can watch an end user interact with the VR environment and evaluate the effectiveness of the tool [27].

For our VR trauma environment, we used an iterative testing process and made changes over 18 times to the setup and VR software. The scenario was tested on a wide demographic, including medical staff, students, residents, developers, research staff, and individuals outside the medical community. We validated the tool through pre- and posttests, and independent raters evaluated medical trainee performance.

#### Look for VR Simulation Champions

With any new technological innovation, it is important to find interprofessional champions to advocate for the adoption of the VR environment [21]. These individuals can help recruit medical trainees, integrate sessions into the curriculum, and engage administrators and clinical colleagues.

Through experience, we would recommend involving program directors, clinical administrators, medical educators, and other health care providers interested in advocating for the adoption of the technology. Clinician investigators conducting research using virtual and augmented reality are another valuable resource. They can provide resources and tips on ways to implement the VR environment more widely in the hospital and medical education curriculum.

Our VR simulation champions were program directors, site leads and investigators, residents, medical students, and the anesthesia research team. Furthermore, since our use case was filling a gap for trauma physicians and anesthesiologists, they became champions to help incorporate our VR environment into the curriculum.

#### Identify Barriers

With the implementation of any innovation, challenges related to technical and nontechnical factors need to be considered. Teams should be ready to adapt or switch technologies based on uncovered restraints from a technical standpoint. VR glitches should be carefully documented and relayed to developers and animators involved in the project. One must also monitor for adverse side effects related to the VR environment, including motion sickness, nausea, dizziness, and headache [28].

From a nontechnical standpoint, there can be challenges related to the logistics and adoption of the VR environment. One concern involves determining who will finance the VR program and which health care team members will have access to the environment. Some basic considerations, such as where the equipment will be stored, who is responsible for cleaning and charging the equipment between uses, and how users will book VR training sessions should be determined. On a larger scale, for VR-based simulation to be used effectively, the setup and assessments must be standardized and reproducible. We recommend organizing training tutorials with both end users and facilitators and carving out dedicated clinical time in the medical curriculum.

The technical challenge that we faced was switching from a bulky VR headset that required connection to a gaming laptop and sensors on tripods to a stand-alone VR headset. This transition allowed us to run the scenario on the VR headset itself. On the nontechnical side, we used the simulation center and hospital research department as the hub for the VR program.

#### Test the Impact of the VR Tool

It is important to validate the impact of the VR tool based on the objectives created previously using an educational evaluation model. Following the Kirkpatrick model [11,12] includes answering questions about reactions (“Did the learners react favorably to the VR environment?”), learning (“Did the learners acquire the intended knowledge and skills?”), behavior (“Did the VR education change behavior?”) and results (“Did the VR education influence clinical performance?”).

With VR, it should be decided which evaluations will be embedded in the VR environment and which will be completed through other means (eg, paper or online questionnaires). The VR tool should undergo utility and usability testing throughout the development process; the tool can also be scrutinized during research studies, such as randomized controlled trials. Through these various evaluation metrics, the VR environment may be regarded by teaching hospitals and medical bodies as a more valuable educational tool and lead to easier uptake.

We assessed the VR airway decision-making scenario through usability testing with developers and through clinical trials with medical students, residents, and physicians. Currently, as a group, we are gathering this data to showcase the influence of the VR environment on knowledge acquisition, clinical behavior, and performance.

#### Amplify VR in the 21st Century: Value Within the Broader Curriculum

VR has been shown to be beneficial for anatomy training [8], procedure education [9,10], and clinical decision-making [29]. However, the VR environment should be embedded in the broader medical curriculum and still be supported by grand rounds, quality assurance meetings, e-learning modules, and simulation center visits. These educational tools, coupled with real patient encounters, can lead to the next generation of clinically competent health care members.

It is the responsibility of the interprofessional team to ensure that supplemental resources, such as prebriefings and assessments, are available for the medical trainee, as this will allow for greater implementation of the VR environment within the medical curriculum. During the global pandemic, where social distancing and remote education present challenges for clinical learning, VR enables medical trainees to continue participating in engaging and interactive training. Importantly, VR can also be incorporated in underresourced and rural communities as a supplemental teaching modality.

In our case, we have already begun using the VR airway scenario with medical students and anesthesia residents in our clinical teaching curricula (Multimedia Appendix 1). VR breaks down geographic barriers, which allows us to easily test and implement the environment in other medical education departments around the world.

### Conclusion

Technological advances and VR in health care are beginning to have practical applications in medical education programs. VR is an accessible, standardized, and safe medical tool that allows medical trainees to practice skills without patients or hospital infrastructure. The opportunity to repeatedly practice anywhere without real consequences to a patient is one of the main advantages of VR technology. This aspect, coupled with the minimal resources involved in facilitating a VR environment, is a driving force behind the adoption of this technology in the medical curriculum. The foundation of a successful VR-based medical simulation environment requires a strong interprofessional team to establish the VR objectives, select the VR modality, and cocreate the VR environment. Once a prototype is designed, the VR environment must be tested meticulously and incorporated into the medical curriculum through VR simulation champions. The implementation of VR is challenging, but through this tutorial, we provide educators with a framework (BUILD REALITY) that can be used to design and implement VR-based medical education training in their curricula.

## Appendix

Multimedia Appendix 1 Clip of immersive VR airway trauma scenario using the Oculus Quest (source: www.chisil.ca).

Multimedia Appendix 2 Learning objectives created for the virtual reality airway trauma study.

Multimedia Appendix 3 Key steps and decision-making tree in the virtual reality airway trauma environment to successfully complete the simulation.

## Abbreviations

BUILD REALITY: begin, use, identify, leverage, define, recreate, educate, adapt, look, identify, test, amplify
VR: virtual reality

## References

[1] Zendejas B, Wang AT, Brydges R, Hamstra SJ, Cook DA (2013). Cost: the missing outcome in simulation-based medical education research: a systematic review. Surgery.

[2] Savoldelli GL, Naik VN, Hamstra SJ, Morgan PJ (2005). Barriers to use of simulation-based education. Can J Anaesth.

[3] Zhou N, Deng Y (2009). Virtual reality: A state-of-the-art survey. Int J Autom Comput.

[4] Ruthenbeck GS, Reynolds KJ (2017). Virtual reality for medical training: the state-of-the-art. J Simul.

[5] Li L, Yu F, Shi D, Shi J, Tian Z, Yang J, Wang X, Jiang Q (2017). Application of virtual reality technology in clinical medicine. Am J Transl Res.

[6] Pottle J (2019). Virtual reality and the transformation of medical education. Future Healthc J.

[7] Jimenez YA, Cumming S, Wang W, Stuart K, Thwaites DI, Lewis SJ (2018). Patient education using virtual reality increases knowledge and positive experience for breast cancer patients undergoing radiation therapy. Support Care Cancer.

[8] Kolla S, Elgawly M, Gaughan JP, Goldman E (2020). Medical Student Perception of a Virtual Reality Training Module for Anatomy Education. Med Sci Educ.

[9] Mohamadipanah H, Perrone KH, Nathwani J, Parthiban C, Peterson K, Wise B, Garren A, Pugh C (2019). Screening surgical residents' laparoscopic skills using virtual reality tasks: Who needs more time in the sim lab?. Surgery.

[10] Mao RQ, Lan L, Kay J, Lohre R, Ayeni OR, Goel DP, Sa Darren de (2021). Immersive virtual reality for surgical training: a systematic review. J Surg Res.

[11] Kirkpatrick D (1998). Evaluating Training Programs: The Four Levels. Second Edition.

[12] Paull M, Whitsed C, Girardi A (2016). Applying the Kirkpatrick model: Evaluating an Interaction for Learning Framework curriculum intervention. Issues Educ Res.

[13] Boet S, Bould MD, Layat Burn C, Reeves S (2014). Twelve tips for a successful interprofessional team-based high-fidelity simulation education session. Med Teach.

[14] Shen J, Xiang H, Luna J, Grishchenko A, Patterson J, Strouse RV, Roland M, Lundine JP, Koterba CH, Lever K, Groner JI, Huang Y, Lin ED (2020). Virtual Reality–Based Executive Function Rehabilitation System for Children With Traumatic Brain Injury: Design and Usability Study. JMIR Serious Games.

[15] Brown RKJ, Petty S, O'Malley S, Stojanovska J, Davenport MS, Kazerooni EA, Fessahazion D (2018). Virtual reality tool simulates MRI experience. Tomography.

[16] Mikropoulos TA, Natsis A (2011). Educational virtual environments: A ten-year review of empirical research (1999–2009). Computers & Education.

[17] Makransky G, Petersen GB (2021). The Cognitive Affective Model of Immersive Learning (CAMIL): a Theoretical Research-Based Model of Learning in Immersive Virtual Reality. Educ Psychol Rev.

[18] Haluck RS, Krummel TM (2000). Computers and virtual reality for surgical education in the 21st century. Arch Surg.

[19] Alam F, Matava C (2022). A New Virtual World? The Future of Immersive Environments in Anesthesiology. Anesth Analg.

[20] Bovill C, Cook-Sather A, Felten P, Millard L, Moore-Cherry N (2015). Addressing potential challenges in co-creating learning and teaching: overcoming resistance, navigating institutional norms and ensuring inclusivity in student–staff partnerships. High Educ.

[21] Lövquist Erik, Shorten G, Aboulafia A (2012). Virtual reality-based medical training and assessment: The multidisciplinary relationship between clinicians, educators and developers. Med Teach.

[22] Stachiw S How To Create Original VR Content: Everything You Need To Know. Roundtable Learning.

[23] Conigliaro RL, Peterson KD, Stratton TD (2020). Lack of diversity in simulation technology: an educational limitation?. Simul Healthc.

[24] Teófilo MR, Lourenço AA, Postal J, Silva YM, Lucena VF (2019). The Raising Role of Virtual Reality in Accessibility Systems. Procedia Comput Sci.

[25] Page-Cutrara K, Turk M (2017). Impact of prebriefing on competency performance, clinical judgment and experience in simulation: An experimental study. Nurse Educ Today.

[26] Nielsen J (1994). Usability Engineering.

[27] Tsai T, Harasym P, Nijssen-Jordan C, Jennett P, Powell G (2003). The quality of a simulation examination using a high-fidelity child manikin. Med Educ.

[28] Regan C (1995). An investigation into nausea and other side-effects of head-coupled immersive virtual reality. Virtual Real.

[29] Mantovani F, Castelnuovo G, Gaggioli A, Riva G (2003). Virtual reality training for health-care professionals. Cyberpsychol Behav.

