# Supplementary material for: Creating a Successful Virtual Reality–Based Medical Simulation Environment: Tutorial
Source: JMIR Med Educ. 2023 Feb 14;9:e41090. doi: 10.2196/41090 (PMC9975916; doi:10.2196/41090)
Supplement: Multimedia Appendix 2 [file mededu_v9i1e41090_app2.pdf]

LEARNING OBJECTIVES

1. Airway injuries are rare, yet significant, considerations in any trauma involving the head or neck.
2. These injuries can be broadly divided into three distinct anatomical categories: maxillofacial, neck, and laryngeal injury.
3. Similarly, they can be divided into two distinct etiological categories: blunt and penetrating traumas.
4. Airway management always begins with an assessment of the airway, which includes removing any potentially obstructing material and administering 100% oxygen.
5. The physical exam in the context of traumatic airway injury must include looking inside the patient’s mouth for obstruction, carefully examining the head and neck for bruising, and palpating for subcutaneous emphysema.
6. If a definitive airway is required to stabilize a patient with an airway injury, great caution must be taken to avoid worsening the injury (particularly with methods of indirect intubation). Videolaryngoscopy and fibre optic bronchoscopy are therefore critical tools in the management of airway injury.
