# Supplementary figures and images for: Creating a Successful Virtual Reality–Based Medical Simulation Environment: Tutorial
Source: JMIR Med Educ. 2023 Feb 14;9:e41090. doi: 10.2196/41090 (PMC9975916; doi:10.2196/41090)

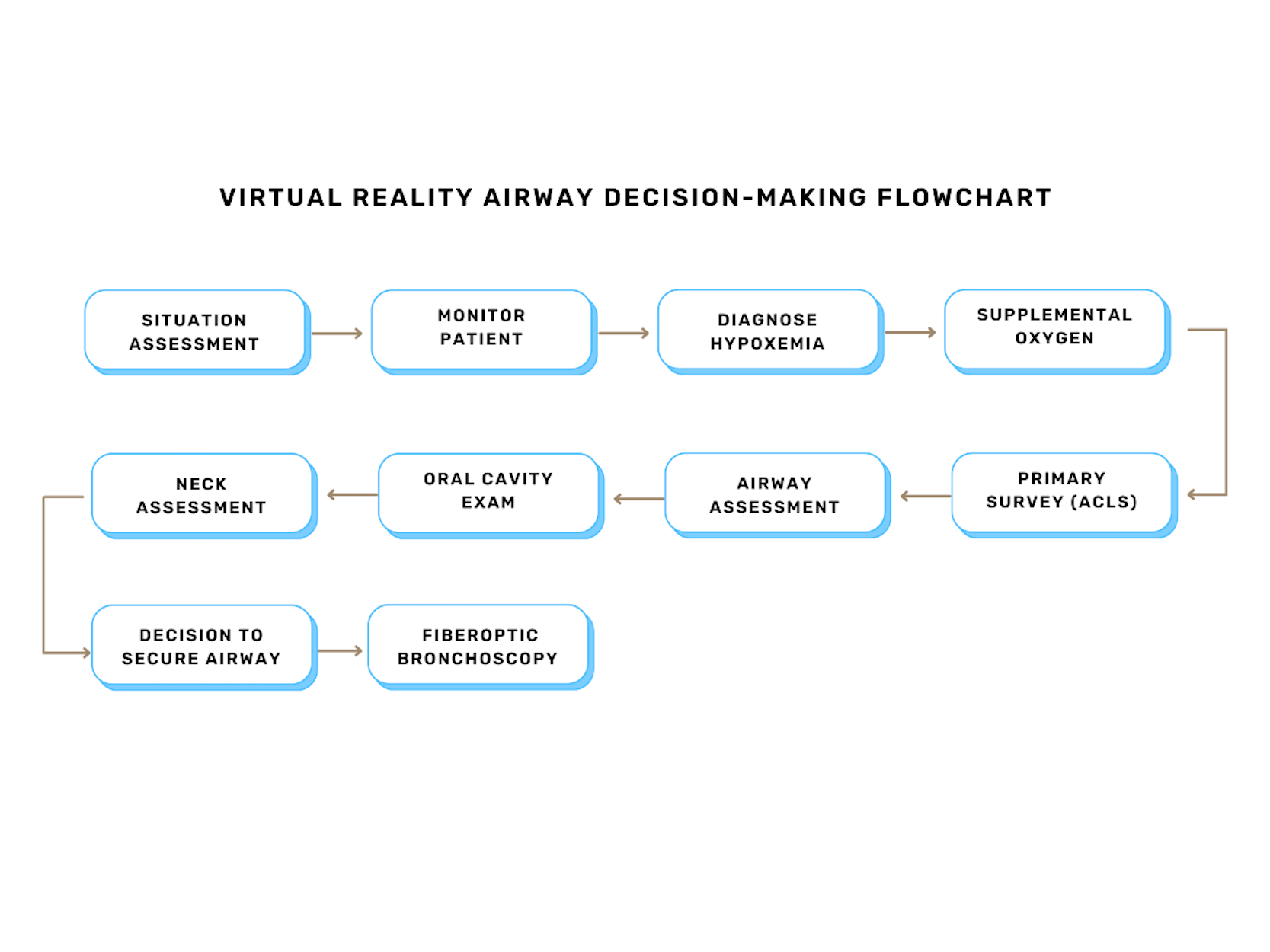

Supplement: Multimedia Appendix 3 [file mededu_v9i1e41090_app3.png]
